# A Simple and Accurate Model to Predict Responses to Multi-electrode Stimulation in the Retina

**DOI:** 10.1371/journal.pcbi.1004849

**Published:** 2016-04-01

**Authors:** Matias I. Maturana, Nicholas V. Apollo, Alex E. Hadjinicolaou, David J. Garrett, Shaun L. Cloherty, Tatiana Kameneva, David B. Grayden, Michael R. Ibbotson, Hamish Meffin

**Affiliations:** 1 National Vision Research Institute, Australian College of Optometry, Carlton, Victoria, Australia; 2 Department of Electrical and Electronic Engineering, NeuroEngineering Laboratory, University of Melbourne, Parkville, Victoria, Australia; 3 Department of Physics, University of Melbourne, Parkville, Victoria, Australia; 4 Department of Optometry and Vision Sciences, ARC Centre of Excellence for Integrative Brain Function, University of Melbourne, Parkville, Victoria, Australia; 5 Centre for Neural Engineering, University of Melbourne, Parkville, Victoria, Australia; The University of Texas at Austin, UNITED STATES

## Abstract

Implantable electrode arrays are widely used in therapeutic stimulation of the nervous system (e.g. cochlear, retinal, and cortical implants). Currently, most neural prostheses use serial stimulation (i.e. one electrode at a time) despite this severely limiting the repertoire of stimuli that can be applied. Methods to reliably predict the outcome of multi-electrode stimulation have not been available. Here, we demonstrate that a linear-nonlinear model accurately predicts neural responses to arbitrary patterns of stimulation using in vitro recordings from single retinal ganglion cells (RGCs) stimulated with a subretinal multi-electrode array. In the model, the stimulus is projected onto a low-dimensional subspace and then undergoes a nonlinear transformation to produce an estimate of spiking probability. The low-dimensional subspace is estimated using principal components analysis, which gives the neuron’s electrical receptive field (ERF), i.e. the electrodes to which the neuron is most sensitive. Our model suggests that stimulation proportional to the ERF yields a higher efficacy given a fixed amount of power when compared to equal amplitude stimulation on up to three electrodes. We find that the model captures the responses of all the cells recorded in the study, suggesting that it will generalize to most cell types in the retina. The model is computationally efficient to evaluate and, therefore, appropriate for future real-time applications including stimulation strategies that make use of recorded neural activity to improve the stimulation strategy.

## Author Summary

Implantable multi-electrode arrays (MEAs) are used to record neurological signals and stimulate the nervous system to restore lost function (e.g. cochlear implants). MEAs that can combine both sensing and stimulation will revolutionize the development of the next generation of devices. Simple models that can accurately characterize neural responses to electrical stimulation are necessary for the development of future neuroprostheses controlled by neural feedback. We demonstrate a model that accurately predicts neural responses to concurrent stimulation across multiple electrodes. The model is simple to evaluate, making it an appropriate model for use with neural feedback. The methods described are applicable to a wide range of neural prostheses, thus greatly assisting future device development.

## Introduction

Implantable electrode arrays are widely used in clinical studies, clinical practice and basic neuroscience research and have advanced our understanding of the nervous system. Implantable electronic devices can be used to record neurological signals and stimulate the nervous system to restore lost functions. Sensing electrodes have been used in applications such as brain-machine interfaces [1] and localization of seizure foci in epilepsy [2]. Stimulating electrodes have been used for the restoration of hearing [3], sight [4,5], bowel control [6], and balance [7], and in deep brain stimulation (DBS) to treat a range of conditions [8]. Most neuroprostheses operate in an open-loop fashion; they require psychophysics to tune stimulation parameters. However, devices that can combine both sensing and stimulation are desirable for the development of a new generation of neuroprostheses that are controlled by neural feedback. Feedback in neuroprostheses is being explored in applications such as DBS for the enhancement of memory [9], abatement of seizures [10], control of Parkinson’s disease [11], and the control of brain machine interfaces [12].

Models that can accurately characterize a neural system and predict responses to electrical stimulation are beneficial to the development of improved stimulation strategies that exploit neural feedback. Volume conductor models are typically used to describe retinal responses to electrical stimulation, however these are computationally intensive and can be difficult to fit to neural response data [13–15]. Simpler models that can be constrained using neural recordings are necessary for real-time applications. Linear-nonlinear models based on a spike-triggered average (STA) have been successfully used to characterize retinal responses to light [16–19]. Models that incorporate higher dimensional components identified through a spike-triggered covariance (STC) analysis have been explored to describe higher order excitatory and suppressive features of the visual system [20–25]. Generally, STA and STC models make use of white noise inputs and have the advantage that a wide repertoire of possible inputs patterns can be explored. White noise models have previously been explored to describe the temporal properties of electrical stimulation in the retina [26,27]. Spatial interactions between stimulating electrodes has not been previously investigated. An example of a stimulation algorithm that could benefit from an accurate description of the spatial interactions is current steering, which attempts to improve the resolution of a device by combining stimulation across many electrodes to target neurons at a particular point [28].

Two benefits obtained by using neural feedback algorithms are (1) the accurate prediction of the response to an arbitrary stimulus across the electrode array and (2) the ability to fit the device to individual patients from the recorded neural responses to a set of stimuli presented in a reasonable amount of time. Here, we combine whole cell patch clamp recordings from individual retinal ganglion cells (RGCs) with stimulation using a multi-electrode array to demonstrate a model with the above advantages. We find that a simple linear-nonlinear model accurately captures the effects of multi-electrode interactions and estimates the spatial relationship between stimulus and response. The approach is scalable to a large number of electrodes, which is prohibitive to accomplish with psychophysics. In contrast to conventional volume conductor models of electrical stimulation [13–15], our model is straightforward to constrain using neural response data and is orders of magnitude more computationally efficient, making it suitable for use in real-time applications.

## Materials and Methods

### Retinal whole mount preparation

Methods conformed to the policies of the National Health and Medical Research Council of Australia and were approved by the Animal Experimentation Ethics Committee of the University of Melbourne (Approval Number: 1112084). Data were acquired from retinae of Long-Evans rats ranging from 1 to 6 months of age. Long-Evans rats were chosen for several reasons. First, rat RGC morphological types have been examined in detail [29,30] and have similarities to RGCs found in other species, including the macaque monkey [31] and cat [32,33]. Second, the size of the rat retina is larger than the mouse retina and so we were able to cover the entire stimulating electrode array with half of the retina.

**Fig 1:**
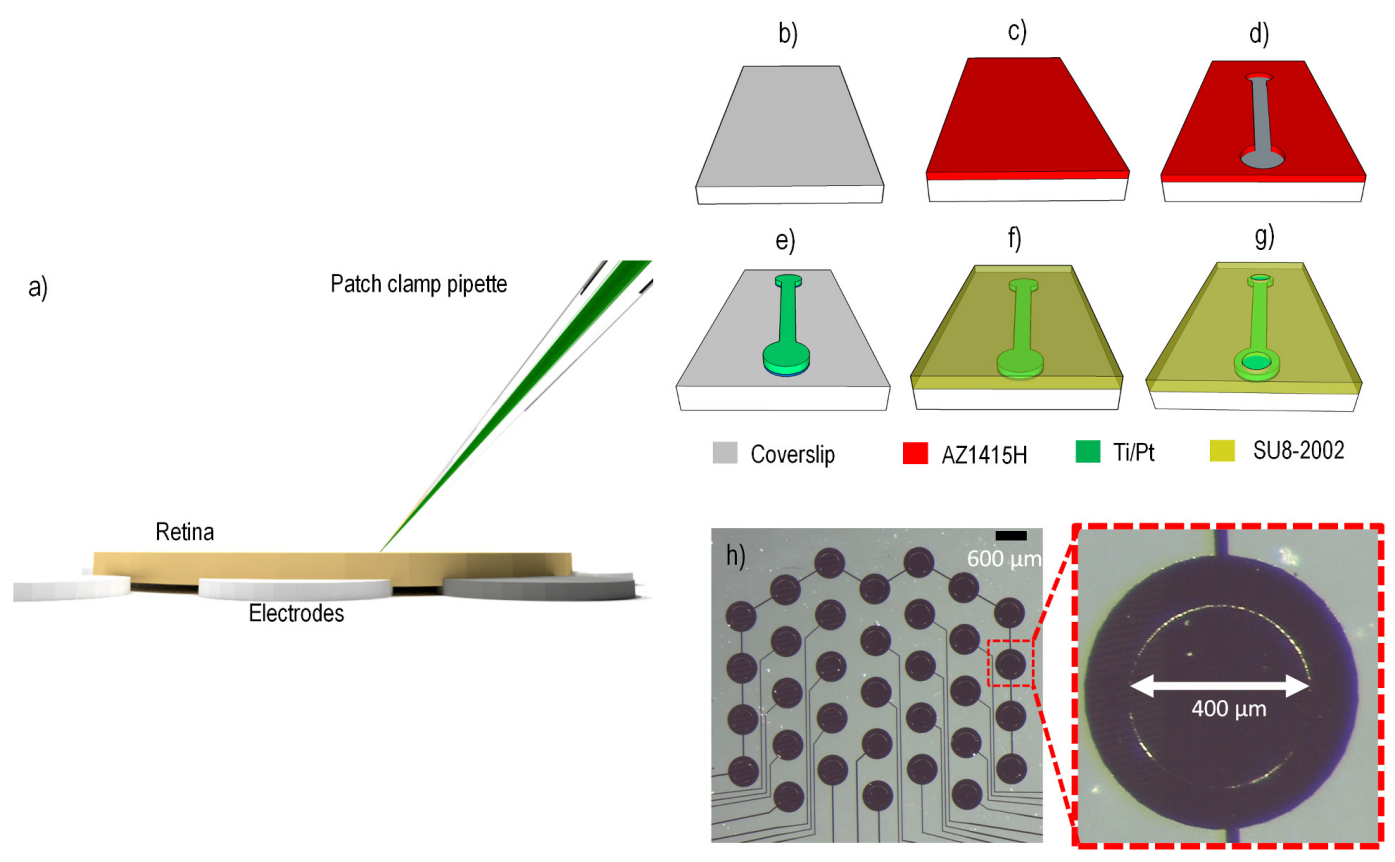
Whole mount preparation and fabrication steps of the platinum stimulating electrode array. (a) The retina was placed on the stimulating array ganglion cell side up. An intracellular patch pipette was used to record from exposed retinal ganglion cell bodies. b) Plasma-cleaned glass cover slip. (c) Glass cover slip spin-coated with positive photoresist. (d) Photoresist removed selectively (dumb-bell shaped pattern for demonstration only) using photolithography mask. (e) Titanium/platinum metal evaporation to create pattern where photoresist from (d) was removed; the residual photoresist was removed using acetone. (f) An insulating layer of SU-8 is spin-coated onto the platinum pattern. (g) The insulation is opened to reveal platinum electrodes having 400 μm diameter. (h) The platinum electrode array is surrounded by a ring of shorted electrodes, which was not used in this work. All electrodes have an exposed disc of 400 μm diameter and a 1 mm center-to-center pitch in the vertical direction. The central portion of the stimulating array (excluding the outer ring) covered an area of approximately 3.5 mm x 3.5 mm.

The animals were initially anesthetized with a mixture of ketamine and xylazine prior to enucleation. After enucleation, the rats were euthanized with an overdose of pentobarbital sodium (350 mg intracardiac). Dissections were carried out in dim light conditions to avoid bleaching the photoreceptors. After hemisecting the eyes behind the ora serrata, the vitreous body was removed and each retina was cut into two pieces. The retinae were left in a perfusion dish with carbogenated Ames medium (Sigma) at room temperature until used. Pieces of retina were mounted on a multi-electrode array (MEA) with ganglion cell layer up and were held in place with a perfusion chamber and stainless steel harp fitted with Lycra threads (Warner Instruments) (Fig 1A). Once mounted in the chamber, the retina was perfused (4–6 mL/min) with carbogenated Ames medium (Sigma-Aldrich, St. Louis, MO) at room temperature. The chamber was mounted on the stage of an upright microscope (Olympus Fluoview FV1200) equipped with a x40 water immersion lens and visualized with infrared optics on a monitor with x4 additional magnification.

### Multi-electrode array fabrication

Electrical stimulation was applied subretinally through a custom-made MEA fabricated on a glass coverslip consisting of 20 platinum stimulating electrodes (Fig 1). Each electrode had an exposed disc of 400 μm diameter, and a vertical pitch of 1 mm. The stimulating area of the MEA spanned an area of 3.5 x 3.5 mm^2^ (excluding the outer ring which was not used). Glass coverslips were cleaned in an oxygen plasma chamber for 20 minutes (Fig 1B). Next, a positive (UV-removable) photoresist (AZ1415H, Microchemicals) was spin-coated onto the surface at 4000 revolutions per minute for 60 seconds (Fig 1C). A laser-printed chrome on soda glass photolithography mask was used to expose a pattern in the photoresist, then developed chemically (MIF726, Microchemicals) revealing openings for electrode pads and tracks (Fig 1D and 1E). The developed cover slips were loaded into an electron beam deposition chamber (Thermionics) and pumped to a vacuum pressure of 1.5×10^−6^ mbar. A 20 nm titanium adhesion layer was deposited at a rate of 0.2 Å/sec, followed by a platinum deposition of 130 nm at a rate of 0.6 Å/sec. Residual photoresist was removed by soaking in acetone for 30 minutes, rinsing with deionized water, and finally oxygen plasma cleaning for 10 minutes. For electrode isolation, a negative (UV-curing) photoresist (SU8-2002, Microchemicals) was spin-coated onto the coverslip and exposed through a different photolithography mask leaving only metal exposed for stimulating electrodes and contact pads (Fig 1F and 1G). The entire device was then cured at 200°C on a temperature-controlled hotplate.

### Data acquisition

Whole cell intracellular recordings were obtained using standard procedures [34] at room temperature. The main reason for recording at room temperature was to ensure that recordings lasted for many hours. To obtain a whole cell recording, a small hole was made in the inner limiting membrane to expose a small number of RGC somas. A pipette was then filled with internal solution containing (in mM) 115 K-gluconate, 5 KCl, 5 EGTA, 10 HEPES, 2 Na-ATP, 0.25 Na-GTP (mosM = 273, pH = 7.3), Alexa Hydrazide 488 (250 mM), and biocytin (0.5%) (Fig 1A). Initial pipette resistance in the bath ranged between 5–10 MΩ. Prior to recording, the pipette voltage was nulled, pipette resistance was compensated with the bridge balancing circuit of the amplifier, and capacitance was compensated on the amplifier head stage. Voltage recordings were collected in current clamp mode and amplified (SEC-05X, NPI electronic), digitized with 16-bit precision at 25 kHz (National Instruments BNC-2090), and stored for offline analysis.

Intracellular recordings lasting up to 4 hours were obtained. Stimulation artefacts that were present in the intracellular recording were removed offline by setting the membrane potential to a constant value for the duration of the stimulus. Spikes in the remaining membrane potential waveform could be easily detected by finding peaks that crossed a set value. Spike times were calculated as the time that the action potential reached its peak value. Spike delay times were calculated by taking the difference between the spike time and the preceding stimulus offset time.

Intrinsic physiological differences, such as spike width, membrane time constant, and input resistance, among RGC types have been described [35,36], which could lead to differences in response latencies to electrical stimulation. Therefore, we performed a k-means cluster analysis on the spike latency from stimulation offset time. The number of clusters (k) to fit was set manually by visual inspection of the clusters. From the cluster analysis, we could detect if there were two or more clusters that might be attributed to direct activation or indirect activation via activation of presynaptic neurons. Unless otherwise stated, responses to electrical stimulation were evaluated by analyzing the short-latency responses. Short-latency responses were spikes that fell within two standard deviations of the mean of the shortest-latency cluster. Long-latency responses fell within the cluster that occurred directly after the short-latency response.

### Electrical stimulation

**Fig 2:**
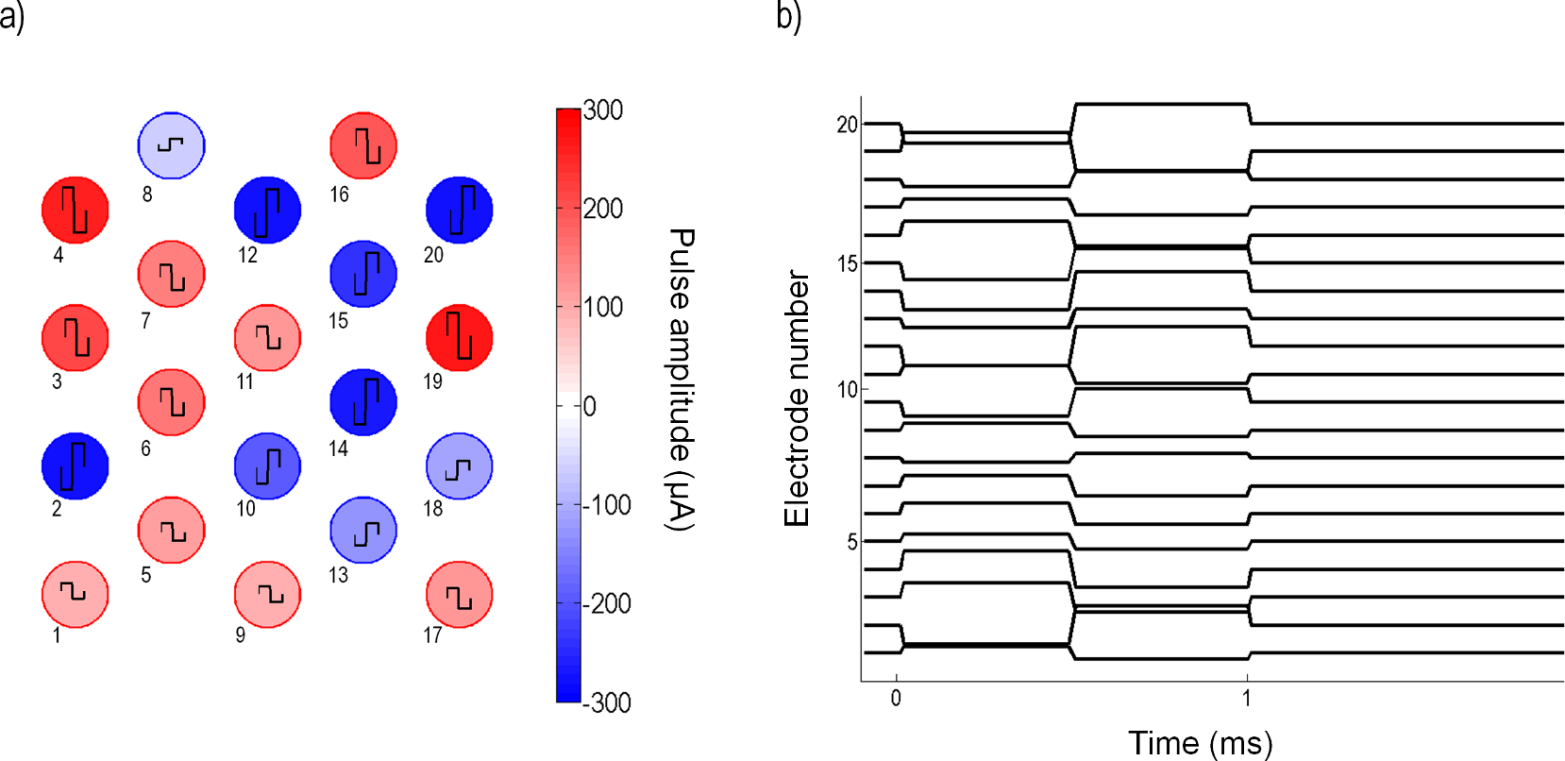
Sample of the random pulses applied to 20 electrodes at a given time. (a) Snapshot of the random amplitude of biphasic pulses applied to all electrodes; the colors indicate amplitudes in μA. A positive amplitude produces an anodic-first pulse; a negative amplitude produces a cathodic-first pulse. Electrode amplitudes were sampled from a Gaussian distribution with variance σ^2^. Electrode numbers are shown below each electrode. (b) Time course of the biphasic pulse applied to each electrode.

Stimulation consisted of biphasic pulses of equal phase duration (500 μs) with an interphase gap (50 μs) and random amplitude. The random amplitudes were sampled from a Gaussian distribution with variance σ^2^. Fig 2 illustrates the random amplitude pulses applied to all electrodes. Stimulation waveform signals were generated by a custom-made MATLAB (MathWorks version 2014a) interface commanding a multi-channel stimulator (Tucker Davis Technologies: RZ2 base station and IZ2 multichannel stimulator). All stimulus amplitudes were bounded by the limits of the stimulator (±300 μA). Biphasic pulses were applied to all electrodes at a frequency of 10 Hz and the numbers of short-latency responses were recorded.

To avoid overstimulation of a cell, an appropriate value of σ was chosen for each cell. Three stimulus trains of 500 pulses were initially generated with fixed σ = 50 μA and applied to the tissue. Next, a new set of stimulus trains were generated using a σ that varied between 50 μA and 250 μA in steps of 50 μA. The number of spikes detected within 5 ms from the stimulus time was used to compute a response probability. A sigmoidal curve was fit to the data of σ versus response probability to find the value of σ that resulted in half the maximum level of response. For cells where the maximum response probability was close to 1, σ was chosen to be a value that resulted in a response probability of 0.5. For other cells that saturated at a response probability less than 1, σ was a lower value.

Once an appropriate value for σ was chosen for the cell, a stimulus vector, $\vec{S_{t}}$, of length 20 (equal to the number of electrodes) was generated by sampling each element from a Gaussian distribution. If the amplitude of stimulation of an electrode exceeded the stimulator limits (±300 μA), then the amplitude value was discarded and a new value was generated from the distribution. Each stimulus was applied 3–5 times before a new $\vec{S_{t}}$ was generated. The experiment continued for as long as the cell’s responses remained stable (usually 3–4 hours). Once cells started to show signs of deterioration (e.g. unstable high frequency spontaneous activity), the experiment was terminated.

### Immunocytochemistry and morphological identification

After recording, the retinal tissue was removed from the chamber and mounted onto filter paper. The tissue was then fixed for ~45 min in phosphate-buffered 4% paraformaldehyde and stored for up to 2 weeks in 0.1 M phosphate-buffered saline (PBS; pH 7.4) at 4°C. The tissue was then immersed in 0.5% Triton X-100 (20 μg/ml streptavidin conjugated Alexa 488; Invitrogen) in PBS overnight to expose biocytin-filled cells. Tissue was washed thoroughly in PBS, mounted onto Superfrost+ slides, and coverslipped in 60% glycerol. Cells were then reconstructed in 3D using a confocal microscope (FluoView FV1200).

RGC types were initially identified by their focal light response at the beginning of each experiment. ON cells showed an increase in spike rate at the onset of light; OFF cells showed an increase in spike rate at the offset of light; ON-OFF cells showed an increase in spike rate at the onset or offset of light. Additionally, RGC classification was correlated with morphology based on dendritic stratification in the inner plexiform layer (IPL) [29,30]. The level of stratification was defined as 0–100% from the level of the inner nuclear layer to the level of the ganglion cell layer. The stratification depth (*s*(*x*)) was quantified as a percentage of the inner plexiform layer (IPL) thickness, according to

$$s\left(x\right)=100\left(\frac{L_{\text{s}}-x}{L_{\text{s}}-L_{\text{e}}}\right).$$ (1)

Here, *x* refers to the depth of a terminal dendrite and *L*_s_ and *L*_e_ represent the IPL-GCL border and the GCL-INL border of the inner plexiform layer, respectively, where depth decreases from the ganglion cell layer towards the photoreceptor layer. Cells that stratified in the inner part of the IPL (*s*(*x*) ≤ 40%) are denoted as OFF-cells. Cells that stratified in the outer part of the IPL (*s*(*x*) ≥ 60%) are referred to as ON-cells. For all cells in this study, the physiological and morphological classifications correlated well. Dendritic field sizes were calculated by tracing out a region connecting the dendritic tips of each cell and fitting an ellipse to the region. The major axis of the ellipse was used to estimate the dendritic field size.

### Mathematical model & model estimation

**Fig 3:**
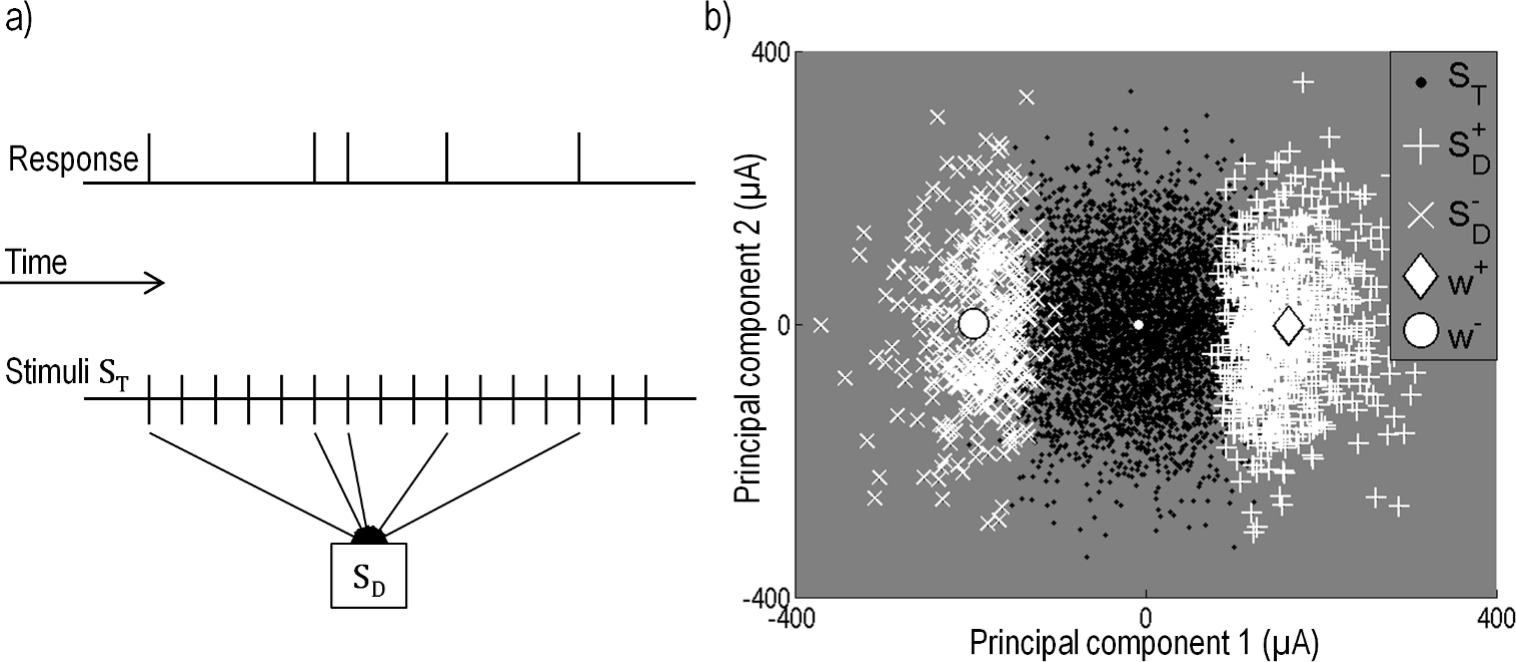
Recovery of the spike-triggered stimuli for the spike-triggered covariance (STC) analysis. (a) Discretized sequence of the neural response and stimulus. Each stimulus consists of a combination of biphasic pulses applied to all 20 electrodes. Stimuli that evoked a spike in the neuron are recorded in the stimulus matrix **S**_D._ (b) STC was conducted on the stimuli generating a response, **S**_D_, to separate the stimulus space into a positive and negative region (+ and ×). The x-axis corresponds to the first eigenvector (${\vec{v}}_{1}$); the y-axis corresponds to the second eigenvector (${\vec{v}}_{2}$). Not all stimuli generated a response in the neuron. Shown in black are the total applied stimuli, **S**_T_, which are overlaid by stimuli **S**_D_ (white crosses). Also shown are the projections of the electrical receptive fields, ${\vec{w}}^{+}$ (large diamond) and ${\vec{w}}^{-}$ (large circle).

Our objective was to find a mathematical description able to accurately capture the spiking probability of RGCs to subretinal stimulation using a MEA. We characterized neural responses using an *N*-dimensional linear subspace of the stimulus space, combined with a nonlinearity describing the intrinsic nonlinear firing properties of neurons. Using STC analysis, we derived the lower dimension stimulation subspace that led to a short-latency response in the neuron. By projecting the raw and spike-triggered stimuli onto the lower dimension subspace, we estimated the intrinsic nonlinearity. The probability of generating a spike, given stimulus $\vec{S_{t}}$, was estimated as

$$P\left(R=spike|\vec{S_{t}}\right)=N_{N}\left({\vec{v}}_{1}∙\vec{S_{t}},{\vec{v}}_{2}\vec{{∙S}_{t}},\ldots ,{\vec{v}}_{N}\vec{{∙S}_{t}}\right),$$ (2)

where N represents the static nonlinear function operating on arguments in μA and ${\vec{v}}_{i}$ (*i* = 1,2,…,*N*) represent the *N* significant principal components. To find ${\vec{v}}_{i}$ (*i* = 1,2,…,*N*), the stimulus data were first separated into a matrix containing only stimuli generating a short-latency response, **S**_D_, and a matrix containing all stimuli, **S**_T_ (Fig 3A). We found the low-dimensional linear subspace that best captured the difference between the spike-triggered stimuli and the raw ensemble by performing principal component analysis (PCA) on the covariance matrix of the spike-triggered ensemble,

$$C_{s}=cov\left(S_{\text{D}}\right),$$ (3)

and comparing it to the variance of the raw ensemble which was approximately σ^2^ in all stimulus directions due to the Gaussian nature of **S**_T_. PCA on *C*_*s*_ produce a set of eigenvectors giving a rotated set of axes in stimulus space and a corresponding set of eigenvalues giving the variance of the spike-triggered ensemble along each of the axes. Eigenvalues that are greater than the variance of the input stimuli reveal the directions where the spike-triggered stimuli have experienced an increase in variance from the raw ensemble. Similarly, eigenvectors that are smaller than the variance of the input stimuli reveal directions where the spike-triggered stimuli have experienced a decrease in variance from the raw ensemble. The eigenvalues that are sufficiently different from the raw ensemble correspond to eigenvectors (${\vec{v}}_{i},i=1,2,\ldots ,N$) pointing in directions in the stimulus space that carry information about the spiking probability of the neuron.

To test if the neural response could be accurately characterized by a one-dimensional model, we examined how many eigenvalues resulting from PCA were significantly different to chance [20]. We compared the eigenvalues obtained by PCA on spike-triggering stimuli to a distribution of eigenvalues for a randomly chosen ensemble of stimuli. This was done by randomly time-shifting the spike train and performing PCA on the corresponding *randomized spike-triggered stimuli* to recover a new set of eigenvalues. By repeating these 1000 times, we construct a distribution of eigenvalues and set a confidence criterion outside of which we presumed the magnitude of the true eigenvalues to be significant. The confidence criterion used was two standard deviations, or a 95% confidence interval. If the greatest or least eigenvalue fell outside these bounds, we rejected the null hypothesis that the spike-triggered ensemble was not significantly different to the full ensemble. We then projected out the axis corresponding to this eigenvalue from the raw data. We repeated the test until all remaining eigenvalues fell within the bounds of the null distribution, suggesting that the remaining eigenvalues were insignificant in affecting the variance of the neuron. Components having an eigenvalue significantly greater than the variance of the randomly time-shifted ensemble were considered to be components that generate an excitatory response on the cell. Conversely, components that are significantly smaller than the variance of the raw ensemble were considered to be components that suppressed the cell’s response.

For the majority of cells, we found that a simplification to one-dimension (${\vec{v}}_{1}$) accurately captured the spike-triggering information, thereby reducing the equation to one dimension. Using this simplification, Eq (2) becomes

$$P\left(R=\text{spike}|\vec{S_{t}}\right)=N_{1}\left({\vec{v}}_{1}\cdot \vec{S_{t}}\right).$$ (4)

Results in the literature indicate that response thresholds to electrical stimulation for some cell types might differ depending on pulse polarity [37]. To explore difference in response to pulse polarity, we allowed the probability to be described by two different nonlinear functions and we found the electrical receptive fields (ERFs) for stimuli having a net effect that was either cathodic-first or anodic-first. Eq (4) then becomes

$$P\left(R=\text{spike}|\vec{S_{t}}\right)=N^{+}\left({\vec{w}}^{+}\cdot \vec{S_{t}}\right)+N^{-}\left({\vec{w}}^{-}\cdot \vec{S_{t}}\right)+c{\text{R}}_{\text{s}},$$ (5)

where $N^{+}$ and $N^{-}$ represent static nonlinear functions and ${\vec{w}}^{+}$ and ${\vec{w}}^{-}$ represent the ERFs for stimuli with positive projections (${\vec{v}}_{1}∙\vec{S_{t}}>0$, net anodic-first) and negative (${\vec{v}}_{1}∙\vec{S_{t}}<0$, net cathodic-first), respectively. R_s_ represents the spontaneous firing rate in Hz and *c* represents a scaling factor of units Hz^-1^. To find the nonlinearities and the ERFs, the first principal component (${\vec{v}}_{1}$) was used to divide the stimulus space into positive and negative regions by projecting all stimuli of **S**_D_ and **S**_T_ onto the first principal component (Fig 3B). Positive and negative regions were defined by the stimuli having either a positive or negative projection onto the first principal component. This produced two spike-triggered stimulus matrices, $S_{D}^{+}$ and $S_{D}^{-}$. The means of the matrices are analogous to the spike-triggered average for net anodic-first and net cathodic-first stimuli [16], and provide an estimate of the ERFs, ${\vec{w}}^{+}$ and ${\vec{w}}^{-}$, respectively. Fig 3B shows an example of the stimuli projected onto the first two principal components and the ERFs, ${\vec{w}}^{+}$ and ${\vec{w}}^{-}$. After the stimuli were separated into two regions, the nonlinear functions, $N^{+}$ and $N^{-}$, were recovered by projecting all stimuli onto the normalized vectors ${\vec{w}}^{+}$ and ${\vec{w}}^{-}$ and segmenting the projected stimuli into 30 bins (15 for the net anodic-first and 15 for the net cathodic-first regions) such that each bin contained an equal number of spikes. By comparing the number of spike-eliciting stimuli to the total number of stimuli in each bin, an estimate of the spike probability was generated. The nonlinear function from Eq (5) was then fit to the data, with the following equations for the sigmoidal curves:

$$N^{+}\left(x_{+}\right)=\frac{a_{+}}{1+\text{exp}\left(-b_{+}\left(x_{+}-c_{+}\right)\right)}$$ (6)

$$N^{-}\left(x_{-}\right)=a_{-}-\frac{a_{-}}{1+\text{exp}\left(-b_{-}\left(x_{-}-c_{-}\right)\right)},$$ (7)

where $x_{+}={\vec{w}}^{+}∙\vec{S_{t}}$ and $x_{-}={\vec{w}}^{-}∙\vec{S_{t}}$. Coefficients *a*_+_ and *a*_−_ represent scaling factors that determine the saturation amplitudes, *b*_+_ and *b*_−_ represent the gain of the sigmoidal curves, and *c*_+_ and *c*_−_ represent the thresholds (50% of the saturation level) for the net anodic-first and net cathodic-first stimulation, respectively. Note that the vectors ${\vec{w}}^{+}$ and ${\vec{w}}^{-}$ might not necessarily be parallel to each other, nor parallel to ${\vec{v}}_{1}$. This may result in electrodes that differentially influence the neuron’s response to anodic-first or cathodic-first stimulation.

To test the similarity between the positive and negative ERFs, we calculated the correlation coefficient between them. A correlation coefficient close to -1 indicated that the two ERFs are approximately equal in magnitude but opposite in sign, and therefore the cell was equally influenced by the same combination of electrodes. A value closer to 0 indicates that the two ERFs have no correlation, and therefore the cell is not influenced by the same electrodes. Positive correlation coefficients were not expected and did not occur. The spatial extent over which a cell was influenced by electrical stimulation was estimated by computing a weighted mean of the distance between the cell and the electrodes. The distance between the cell and each electrode center was weighted by the electrode’s influence on the cell as given by the ERFs. The weighted mean for both ERFs was given by,

$$D^{+}=\frac{\sum_{i=1}^{20}w_{i}^{+}d_{i}}{\sum_{i=1}^{20}w_{i}^{+}}$$ (8)

$$D^{-}=\frac{\sum_{i=1}^{20}w_{i}^{-}d_{i}}{\sum_{i=1}^{20}w_{i}^{-}}$$ (9)

where $w_{i}^{+}$ and $w_{i}^{-}$ are the weights given by ${\vec{w}}^{+}$ and ${\vec{w}}^{-}$ respectively, and *d*_*i*_ is the distance between the cell and electrode *i*.

To test which electrodes significantly affected the cell's response, ${\vec{w}}^{+}$ and ${\vec{w}}^{-}$ were recalculated 1000 times by projecting the data onto the first eigenvector of the randomly time-shifted distribution of eigenvectors from the significance test. A distribution for ${\vec{w}}^{+}$ and ${\vec{w}}^{-}$ was constructed from which the true ${\vec{w}}^{+}$ and ${\vec{w}}^{-}$ could be compared. Electrodes from the true ${\vec{w}}^{+}$ and ${\vec{w}}^{-}$ were compared to the root mean square (RMS) of the distribution and electrodes that were larger than the RMS bounds were considered significant.

For cells where more than one significant principal component was obtained from the significance test, we compared the variance explained by the first principal component to that of the next most significant component. This was done by comparing the separation of the first eigenvalue *e*_1_ from the mean of the randomized distribution of eigenvalues (${\bar{e}}_{\text{rnd}}$) with the separation between the next most significant eigenvalue (*e*_2_) and the same mean. The strength was defined as

$$G=\frac{\left|e_{1}-{\bar{e}}_{\text{rnd}}\right|}{\left|e_{2}-{\bar{e}}_{\text{rnd}}\right|},$$ (10)

and gives a relative measure of how much larger *e*_1_ is compared to the next most significant eigenvalue. ${\bar{e}}_{\text{rnd}}$ was calculated from the first iteration of the hypothesis test.

### Model validation

For each cell, 80% of the data were used to fit the model parameters, while the remaining data were used to validate the model. To obtain a quantitative estimate of the performance of the model, the probability of response given a stimulus was calculated from the validation data and compared to the model prediction. The validation stimuli were assigned 1 if they produced a direct response and 0 otherwise. Each stimulus was also assigned a predicted probability using the model (Eq (5)) recovered from the training data. The stimuli were then binned into segments in the range of 0 to 1 depending on their predicted probability and an actual probability for each bin was calculated by the fraction of stimuli assigned a 1. The mean square error (*E*^MS^) was then calculated,

$$E^{\text{MS}}=\frac{1}{B}\sum_{i=1}^{B}{\left({\hat{P}}_{i}-P_{i}\right)}^{2},$$ (11)

where *B* is the number of bins, ${\hat{P}}_{i}$ is the predicted probability, and *P*_*i*_ is the calculated probability from the data for a particular bin. For all cells, *B* was equal to 10. The root mean square error (*E*^RMS^) of the model, given by

$$E^{\text{RMS}}=\sqrt{E^{\text{MS}}},$$ (12)

was used as a quantitative measure of the model accuracy.

We also compared the error of a one-dimensional model to that of a two-dimensional model. The two-dimensional spike probability was estimated by

$$P\left(R=\text{spike}|\vec{S_{t}}\right)=N_{2}\left({\vec{v}}_{1}\cdot \vec{S_{t}},{\vec{v}}_{2}\vec{\cdot S_{t}}\right),$$ (13)

where ${\vec{v}}_{2}$ represented the next most significant component, either the second (excitatory) or last (suppressive) principal component. To find the two-dimensional nonlinearity ($N_{2}$), a surface was fit to the spike-triggered data projected onto these two most significant components. The surface fit was obtained using a cubic spline interpolation on MATLAB’s curve fitting toolbox. Once the surface was fit, the validation data was used to calculate the mean model error calculated using Eqs (11) and (12).

## Results

### Stimulation

**Fig 4:**
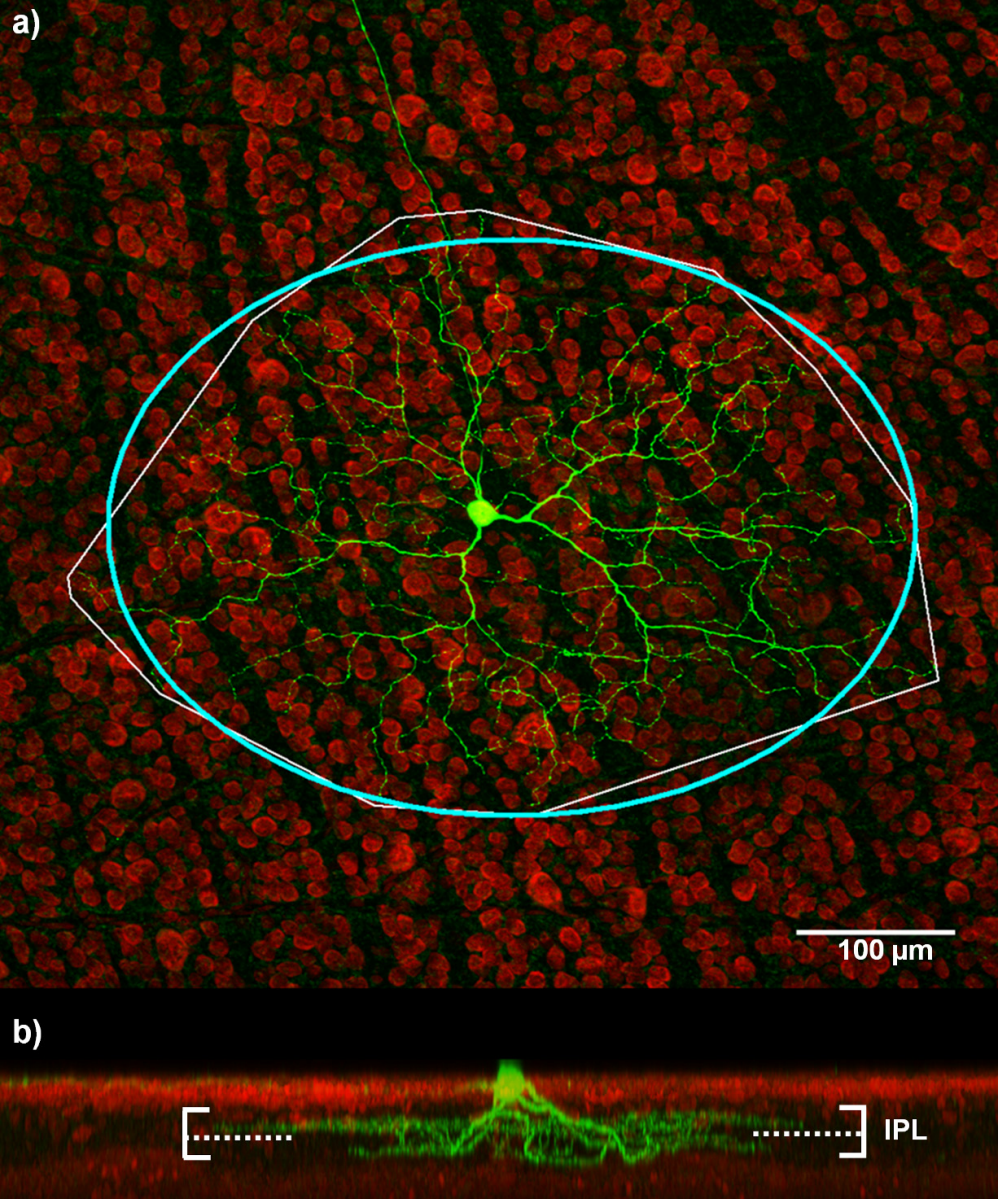
ON-OFF retinal ganglion cell. (a) A stained image of a RGC from which dendritic stratification and dendritic field size can be analyzed. The bounding box (thin white line) shows the region encompassing the dendritic field of the cell. An ellipse was fit to this region (thick blue line) to estimate the dendritic field size. The major axis of the elliptical fit for this cell was 508 μm. (b) Dendritic stratification for this cell is in two distinct layers of the inner-plexiform layer (IPL). Dendrites in the ON sublamina of the IPL stratify at an average depth of 66.9%, while the OFF dendrites stratify at an average depth of 27.2%. The solid horizontal lines represent the edge of the IPL. The dashed line represents the approximate ON/OFF border; the region above the dashed line is the ON layer, the region below is the OFF layer.

Intracellular recordings lasting up to 4 hours were obtained from 25 cells. This population included 7 ON, 13 OFF, 3 ON-OFF, and 2 cells where 3-D morphological reconstructions were not possible. Our comparison of histological and physiological results were consistent with those of Huxlin and Goodchild [29]: ON center cells stratify in the inner IPL (40–100% depth), while OFF center cells stratify in the outer IPL (0–40% depth). ON-OFF types stratify in both the inner and outer layers of the IPL. Fig 4 shows an example of an ON-OFF RGC with dendrites stratifying in both inner and outer layers of the IPL. A summary of the stratification depths for the ON, OFF, and ON-OFF cells are given in Table 1.

**Table 1:** Summary of results.

	Mean	Standard deviation	Min	Max
ON (depth %)	57.1	6.6	49.2	69.6
OFF (depth %)	24.1	9.7	11.0	37.6
ON-OFF (inner) (depth %)	63.8	0.16	57.7	66.9
ON-OFF (outer) (depth %)	20.8	7.7	16.3	27.2
Negative threshold (*c*_−_)	-233	124	-39	-469
Positive threshold (*c*_+_)	235	136	59	416
Average model RMSE	0.064	0.027	0.028	0.117
Mean latency for SL responses (ms) (N = 20)	1.75	1	0.5	4.35
Mean latency for LL responses (ms) (N = 17)	11	2.59	4.2	20.27
1-dimensional nonlinear fit coefficient (r^2^)	0.92	0.04	0.83	0.99

To fit the model parameters, cells were simultaneously stimulated with biphasic pulses on all electrodes, where the amplitude of the pulses were randomly chosen from a Gaussian distribution of zero mean and standard deviation σ (here after *white noise stimuli*). To determine an appropriate value of σ for each cell, three short stimulus trains (approximately 3 min each) of white noise stimuli with different σ were initially presented to the cell (σ varied from 50–250 μA in steps of 50 μA). The number of times the cell responded within 5 ms was used to obtain a response probability. Each cell responded with a different maximum response probability when stimulated with white noise at the highest value of σ; some cells could respond with a spike probability close to one, while others only responded with a spike probability less than one. However, cells that responded to fewer pulses tended to show an increased level of long-latency activity (> 5 ms), most likely due to intensified network activation. The value of σ used for white noise stimulation for each cell in the rest of the experiment was the value corresponding to half the saturation level.

**Fig 5:**
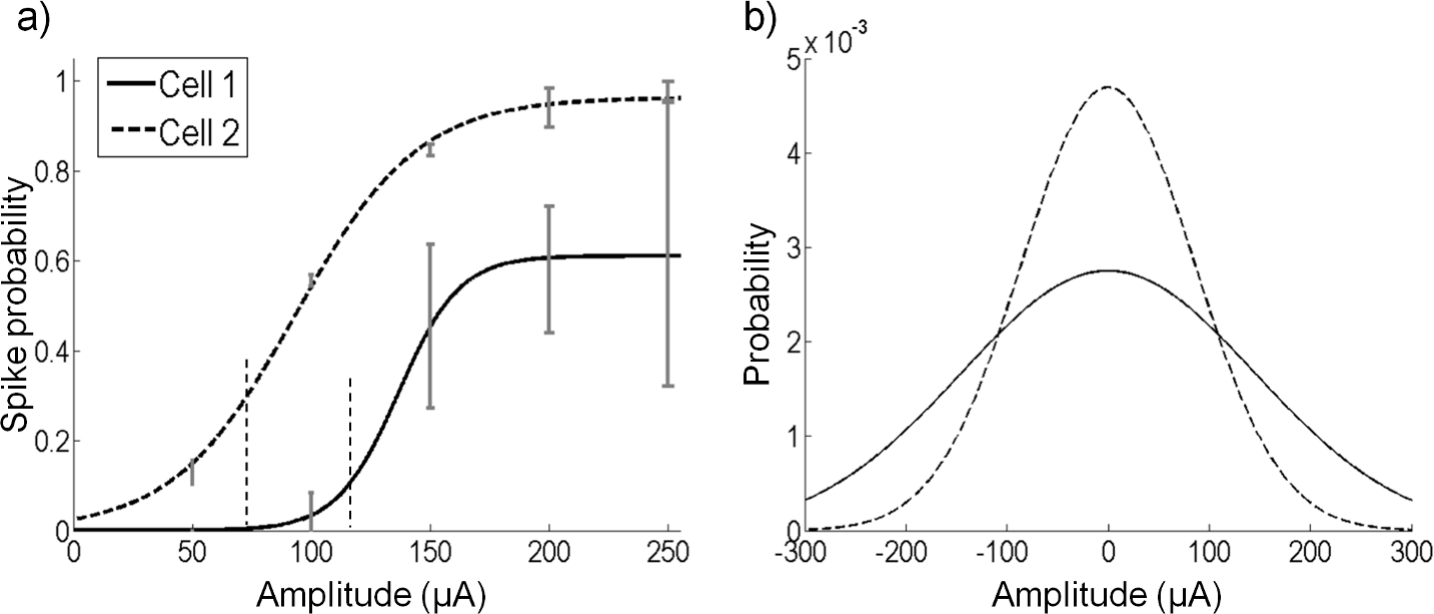
White noise stimulation amplitudes. (a) The increase in spike probability with increasing amplitude of the standard deviation of the white noise for two cells. To avoid over stimulation of recorded cells, white noise stimulation with a standard deviation (σ) was initially applied to each cell. The value of σ corresponding to half the maximal spike probability was used as the standard deviation of white noise stimulation for the experiment. Cell 1 reached a maximum value close to 1. The value of σ corresponding to a 0.5 spike probability was 85 μA. Cell 2 reached a maximal value close to 0.6. The value of σ corresponding to a spike probability of 0.3 was 145 μA. Error bars refer to the standard deviation from the mean. (b) The σ values from (a) were used to generate Gaussian distributions from which stimulation amplitudes on each electrode were sampled. Stimulus amplitudes were limited to ±300 μA, hence the distribution is truncated at these values.

Fig 5 shows examples of two cells with different σ values. Cell 2 responded with a spike probability close to one even at low σ values while cell 1 responded maximally with a spike probability of around 0.6 (Fig 5A). The value of σ used for white noise stimulation for cell 1 was 85 μA and for cell 2 was 145 μA. Note that we used this method to calibrate our experiments and the nonlinear curves do not show the maximum probability of firing, as each point is an average over a variety of stimulus amplitudes. Following this calibration, longer trains of white noise stimulation (approximately 2 minutes each) with the corresponding value of σ for each cell were used to obtain data for recovering the model parameters. The corresponding Gaussian distributions for cells 1 and 2 are shown in Fig 5B. The experiment for each cell lasted approximately 3–4 hours.

**Fig 6:**
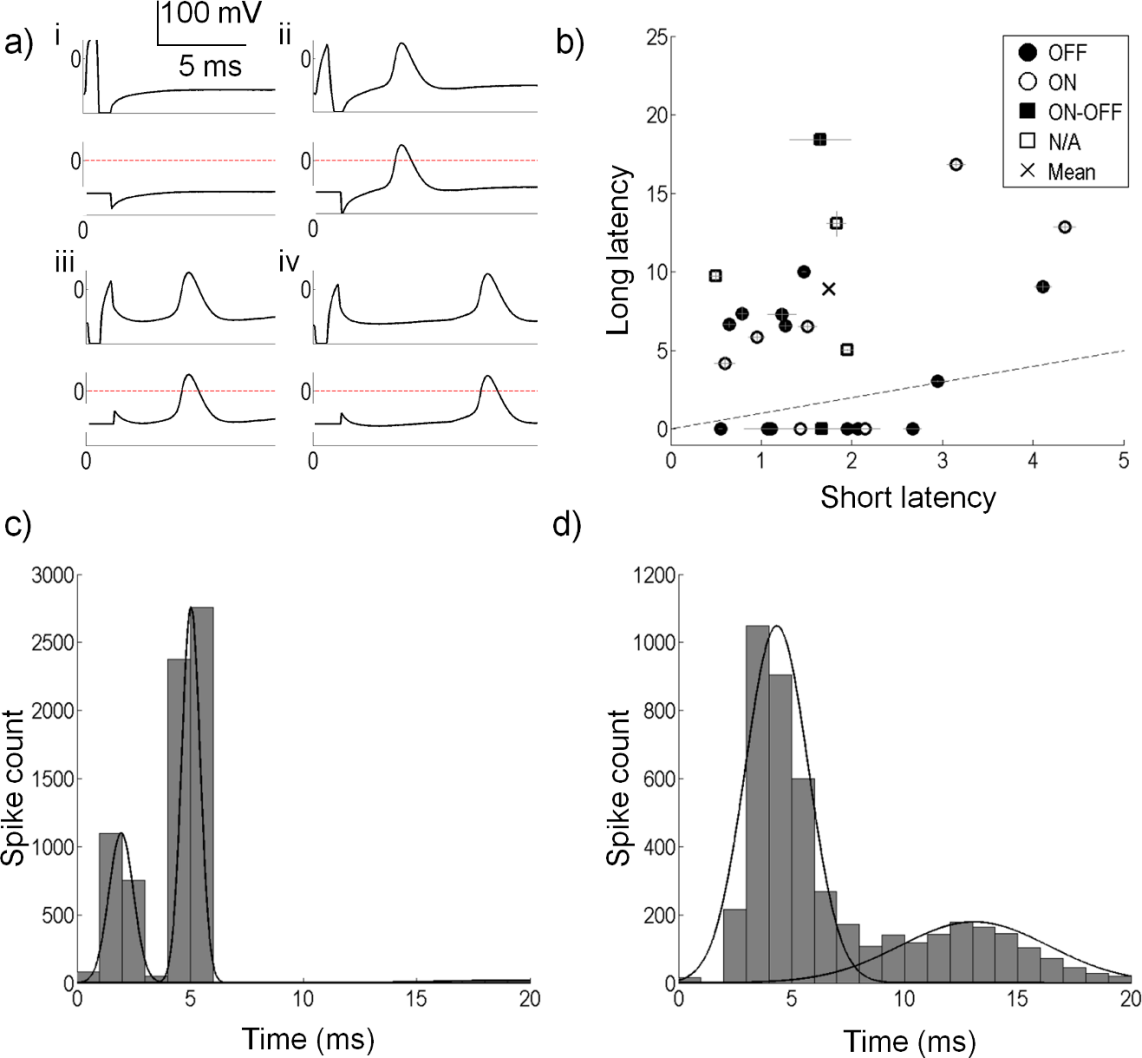
Spike detection and spike latency analysis. (a) Membrane potentials showing the response to four stimulus types; (i) a failed anodic first stimulus, (ii) a successful anodic first stimulus with a short-latency response, (iii) a successful cathodic first stimulus with a short-latency response and (iv) a successful cathodic first stimulus with a long-latency response. Bottom traces in each panel show the stimulus artefact removed by setting the artefact to a constant value, and the threshold used to detect spikes (horizontal line at zero). (b) Short and long spike latencies for all cells. Horizontal lines show the standard error of the short latency clusters. Vertical lines show the standard error for the long latency clusters. Note that for many of the cells, the standard errors are very small. The dashed line shows the line of equality. Eight cells did not produce a long latency component, and hence lie below the dashed line. The × symbol shows the mean of all short and long latencies. (c) Distribution of spike latencies for a sample cell. A k-means cluster analysis (2 clusters) on the spike latencies gives the two distributions shown by the Gaussian distribution. This cell had a short-latency distribution mean of 1.95 ms (SD 0.54 ms). The long-latency distribution mean is 5.03 ms (SD 2.58 ms). (d) Distribution of spike latencies for another sample cell. A k-means cluster analysis (2 clusters) on the spike latencies gives the two distributions shown by the Gaussian distribution. This cell had a short-latency mean of 4.35 ms (SD 1.37 ms). The long-latency distribution mean is 12.87 ms (SD 2.58 ms).

Stimulation artefacts were present in the recordings that could be removed by blanking without affecting the ability to detect the cells’ spikes. Fig 6A shows examples of some of the spiking patterns observed during experiments: (i) a failed anodic-first stimulus, (ii) a successful short-latency anodic-first stimulus, (iii) a successful short-latency cathodic-first stimulus, and (iv) a successful long-latency cathodic-first stimulus. The top panel in each subplot shows the raw recording and the bottom panels show the same signals with the artefact removed by blanking. Also shown in the bottom panels are the thresholds used to detect spikes (horizontal lines). These figures show that spikes could be easily identified without interference from the stimulus artefact.

The spike latencies after a stimulus pulse were analyzed for each cell. Some cells produced a bimodal distribution attributed to the short- and long-latency responses (N = 13), with four cells showing overlapping distributions for the two latencies. The remaining cells only produced short-latency spikes that were close to the timing of the stimulus pulse (N = 8). Fig 6B depicts the spike latencies for all cells. The average short-latency cluster mean for all cells was 1.75 ms from stimulus offset (SD 1 ms). The longest short-latency cluster for a cell had a mean of 4.35 ms (SD 1.37 ms). Fig 6C and 6D show the distributions of spike latencies for two sample cells, along with fitted Gaussian distributions obtained from the cluster analyses. Fig 6C shows a cell with two distinct clusters, with a short-latency cluster mean at 1.95 ms. Fig 6D shows two overlapped clusters with the short-latency cluster mean at 4.35 ms.

### Model estimation

Our aim was to find a mathematical description that could accurately capture the response probability of neurons to concurrent stimulation using a MEA. To do this we first performed a principal components analysis on the ensemble of stimuli that triggered a short latency spike. For all cells we found that the neural response could be well predicted by projection onto a subspace spanned by the first principal component, ${\vec{v}}_{1}$. The variance explained by ${\vec{v}}_{1}$ was significantly higher than that of next greatest component, ${\vec{v}}_{2}$, suggesting that the spiking information was well captured by ${\vec{v}}_{1}$.

**Fig 7:**
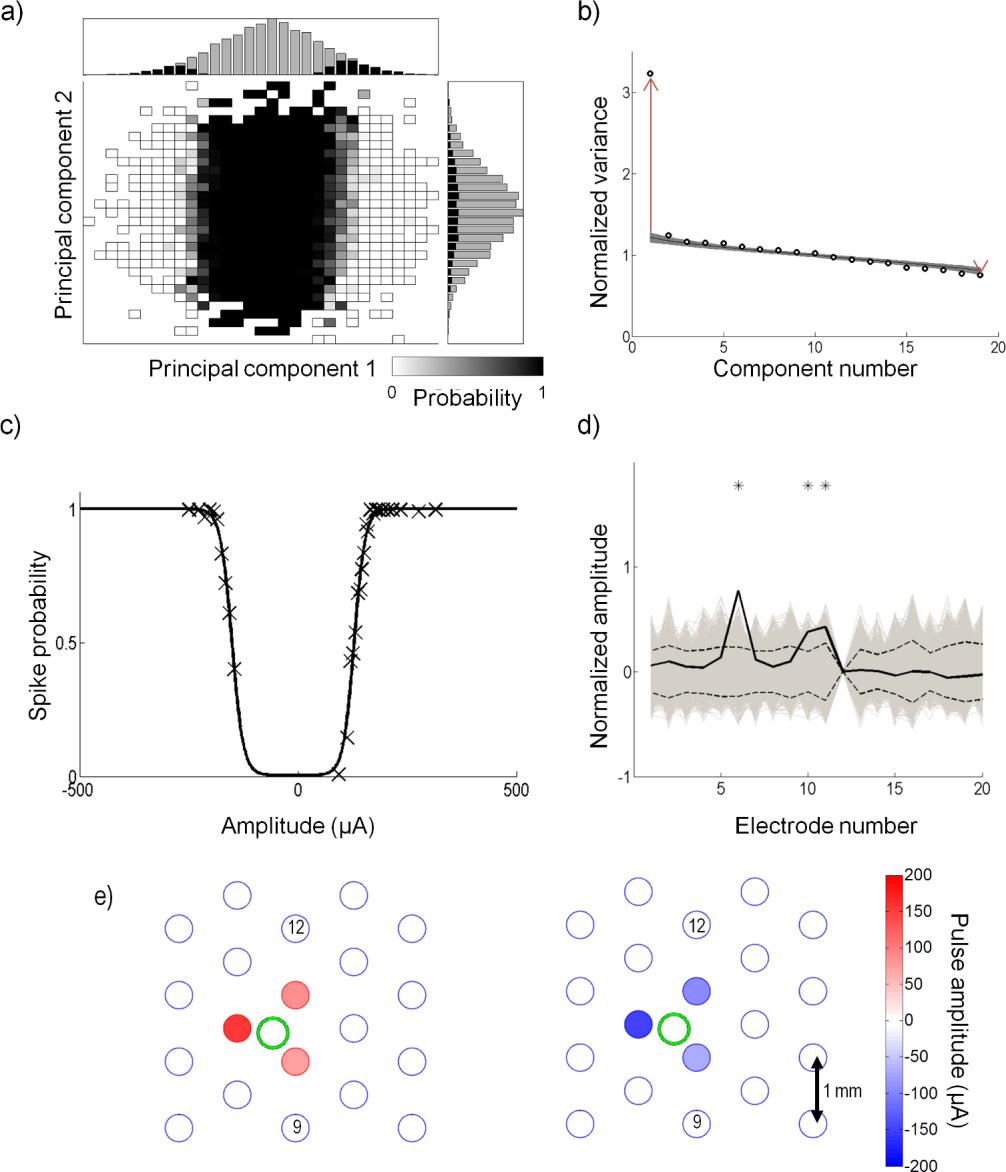
Recovery of model parameters for a sample cell. (a) The stimuli are projected onto the first two principal components, ${\vec{v}}_{1}$ and ${\vec{v}}_{2}$. The grey squares represent the spike probability, where a black value represents 0 probability, and a white value represents a probability of 1. Plotted above and to the right are the histograms of the stimuli (gray) and the spike-triggered stimuli (black) along each component axis. (b) Eigenvalues of the spike-triggered stimuli recovered by principal component analysis (round circles) are plotted. The eigenvalues are normalized by the variance of the input stimuli. The shaded region represents the 95% confidence interval from the statistical hypothesis test. The hypothesis test recovered one significant excitatory and one significant suppressive component. The red arrows show the distance between the two most significant eigenvalues and the mean of the random distribution recovered from the first iteration of the hypothesis test. The length of the arrows represent *d*_1_ and *d*_2_ from Eq (10). (c) The nonlinear function recovered by fitting a double sigmoid to the spike probability projected onto ${\vec{w}}^{+}$ and ${\vec{w}}^{-}$. Open circles represent the raw data and the solid line shows the nonlinear equation fit (r^2^ = 0.98). This cell had a positive and negative threshold (parameters *c*_+_ and *c*_−_ in Eq (5)) of 129 μA and -152 μA proportional to ${\vec{w}}^{+}$ and ${\vec{w}}^{-}$, respectively. (d) The true ${\vec{w}}^{+}$ and ${\vec{w}}^{-}$ (solid black) are compared to the root mean square (dashed line) of a distribution of ${\vec{w}}^{+}$ and ${\vec{w}}^{-}$ (gray). Stars show which electrodes were significant. In this preparation, electrode 12 was not operational. (e) Representation of the amplitudes that generate the ERFs, ${\vec{w}}^{+}$ (left) and ${\vec{w}}^{-}$ (right). The large circles represent the electrode locations. A correlation coefficient of -0.97 was obtained between ${\vec{w}}^{+}$ and ${\vec{w}}^{-}$. Three electrodes significantly affected the cell in ${\vec{w}}^{+}$ and in ${\vec{w}}^{-}$. In this experiment, the retina was placed such that the optic disc was located around electrode 9. The stimulation return electrode was placed distally above electrode 12. The green circle shows the approximate dendritic field of the recorded cell. Stimulus amplitudes ranged up to ±300 μA; however, the range shown here is smaller to make the differences in electrode amplitudes clearer.

Fig 7A shows the spike-triggered probabilities projected onto ${\vec{v}}_{1}$ and ${\vec{v}}_{2}$ from the sample cell in Fig 3B. The histograms show the number of stimuli (gray) and responses (black) along each axis; the ratio of the bars of the two histograms is used to determine the spike probabilities along each axis. From the histograms, it is clear that the distribution of the spike-triggered stimuli was bimodal in the ${\vec{v}}_{1}$ axis; however, it remained unimodal along ${\vec{v}}_{2}$, similar to Gaussian distribution of the full stimulus ensemble.

A statistical hypothesis test was used to determine how many eigenvalues recovered by PCA revealed a significant amount of the spike-eliciting information. The test compares the eigenvalues recovered from the data, to a set of eigenvalues produced by randomly time-shifting the spike train and performing PCA on the new set of stimuli. From the set of time-shifted eigenvalues, a 95% confidence limit was set to determine which eigenvalues from the original spike-triggered data lie outside of the limits. Fig 7B illustrates an example of the hypothesis test. For the sample cell, the test revealed that a large amount of information was contained in the first component, which was excitatory (${\vec{v}}_{1}$, eigenvalue above the confidence interval, long red arrow in Fig 7B). A second suppressive component was also significant, but contained a very small amount of information (the last component, eigenvalue below the confidence interval, short red arrow in Fig 7B). The shaded region shows the 95% confidence intervals from the hypothesis test. The circles represent the eigenvalues obtained from PCA on the spike-eliciting data. After two iterations of the hypothesis test, all eigenvalues were within the 95% confidence intervals. The arrows shown in the figure represent the separation between the mean of the randomly time-shifted distribution of eigenvalues and the raw eigenvalues ($\left|e_{1}-{\bar{e}}_{\text{rnd}}\right|$ and $\left|e_{2}-{\bar{e}}_{\text{rnd}}\right|$ in Eq (10)). For this cell, $\left|e_{1}-{\bar{e}}_{\text{rnd}}\right|$ was approximately 12 times greater than $\left|e_{2}-{\bar{e}}_{\text{rnd}}\right|$ (*G* = 12). Note that for this experiment, electrode 12 was not operational and hence only 19 components were produced.

The bimodal distribution of response probability along the axis of the first principal component indicates that this neuron responded to two categories of multi-electrode stimulation; one category that produced a positive projection onto this axis, and one with a negative projection onto this axis. For all cells, stimuli with a positive projection onto the axis produced a stimulus at the cell’s location whose net effect was anodic-first, regardless of the fact that some electrodes may have been stimulated with cathodic-first pulses. The opposite was true for the negative projection. We therefore wondered if there may be differences in the one-dimensional stimulus subspace to which the cell responded, between net anodic-first and net cathodic-first stimulation. If so, the PCA analysis would only find the average direction of these two one-dimensional subspaces. To address this we used the PCA initial estimate of the subspace to break the stimulus space up into positive and negative regions determined by whether the stimuli had a positive or negative projection onto ${\vec{v}}_{1}$. By separating the data into the two regions, two electrical receptive fields (ERFs), ${\vec{w}}^{+}$ and ${\vec{w}}^{-}$, corresponding to net anodic-first and net cathodic-first stimuli, were estimated (Fig 3B). The ratio of spike-eliciting stimuli to total stimuli was then used to determine a spike probability.

Fig 7C illustrates the spike probability for the sample cell. The raw data was projected onto ${\vec{w}}^{+}$ and ${\vec{w}}^{-}$ (×) and the nonlinear curve from Eq (5) (solid line) was fit to the data. All cells obtained high r^2^ (coefficient of determination) values for the nonlinear fit; the average r^2^ for all cells was 0.92 (SD 0.04) (see Table 1 for summary). This suggests that a double sigmoidal curve is appropriate to describe the nonlinear firing probabilities of RGCs to electrical stimulation.

Significant electrodes in ${\vec{w}}^{+}$ and ${\vec{w}}^{-}$ were determined by comparing the electrodes to a distribution of ${\vec{w}}^{+}$ and ${\vec{w}}^{-}$ generated in the signifcance test. Fig 7D shows the true ${\vec{w}}^{+}$ (solid black line) compared to the RMS of the distribution (dashed line). Up to three electrodes significantly affected this cell. To visualize the cell's ERF, the electrode amplitudes that generated ${\vec{w}}^{+}$ and ${\vec{w}}^{-}$ were plotted. Fig 7E depicts ERFs for the sample cell; the green dot shows the approximate location of the recorded cell soma. The filled circles represent the stimulus amplitudes on the electrode that generate ${\vec{w}}^{+}$ and ${\vec{w}}^{-}$. Only significant electrodes are colored. For all cells, ${\vec{w}}^{+}$ produced a stimulus at the cell location that was anodic-first, while ${\vec{w}}^{-}$ produced a stimulus at the cell location that was cathodic-first. In this example, the retina was oriented such that the optic disc was approximately above electrode 9. ${\vec{w}}^{+}$ and ${\vec{w}}^{-}$ for this cell had a correlation coefficient of -0.91, indicating that the cell was influenced by the same set of electrodes when the stimulus was net anodic or net cathodic-first. An estimate of the size of the ERFs was determined by calculating a weighted mean of the distance between the cell and electrodes, where the distance was weighted by the ERFs. For this sample cell, *D*^+^ and *D*^−^ were approximately equal to 1 mm, which is also the separation of the electrodes.

**Fig 8:**
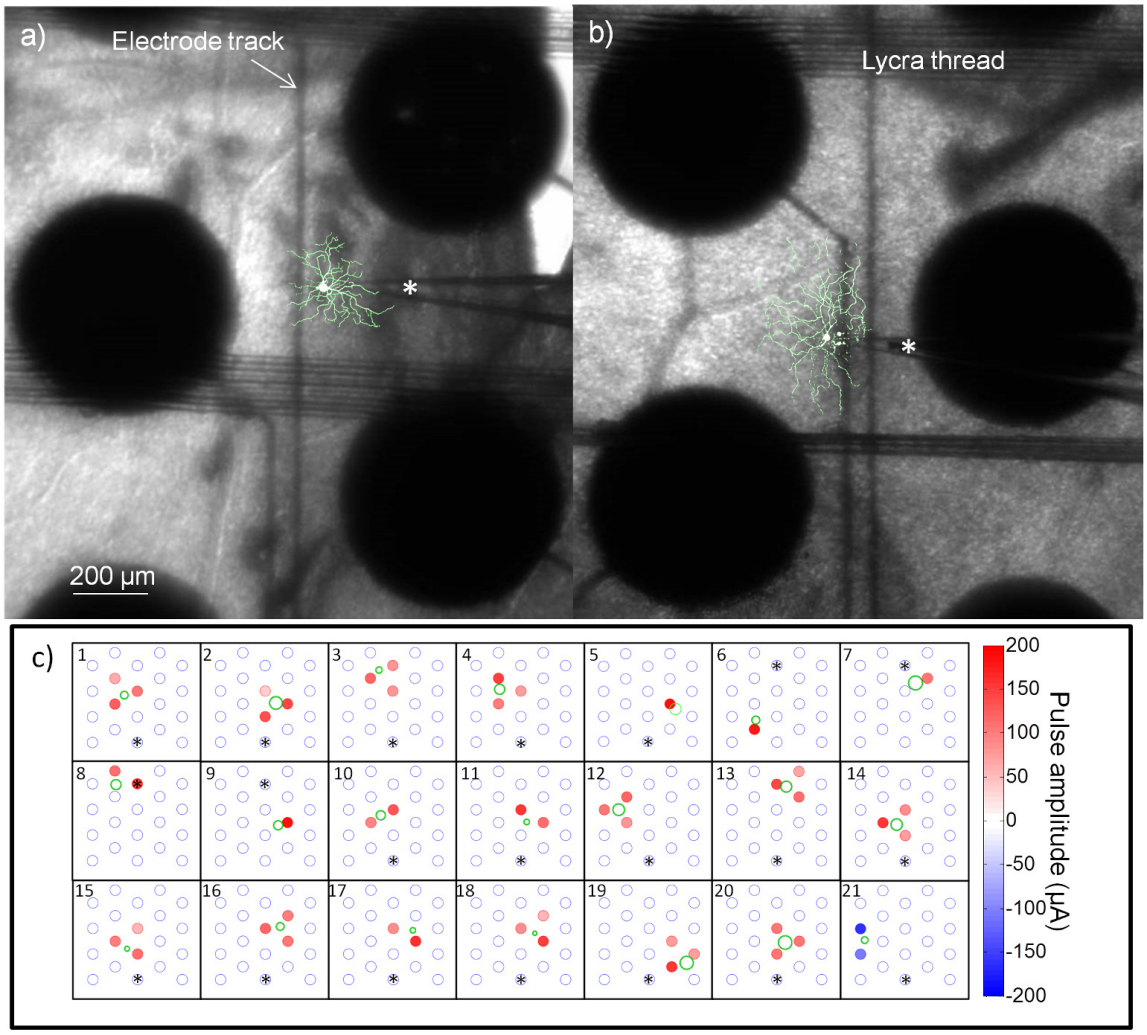
Dendritic and electrical receptive fields. a-b) Sample cells depicting the stimulating array (large black discs) and the patch-clamp recording electrode (denoted by a *). Overlaid on the images are the morphological reconstructions of the cells. The sample cell in (a) is also shown in (c) 16. The sample cell in (b) is also shown in (c) 20. Note that the stimulating electrodes appear large, but the exposed area is only 400 μm. Also visible are the lycra threads used to keep the retina affixed and the stimulating electrode tracks. c) The electrical receptive fields shown together with the dendritic receptive field estimates. The electrodes with stars above them show the approximate location of the optic disc for each preparation.

We investigated the relationship between dendritic receptive field and ERF by comparing the morphological reconstructions to the ERFs obtained from the model. Two sample cells are shown in Fig 8A and 8B. In these images, the morphological reconstruction has been superimposed onto a photograph showing the stimulating array and the patch pipette during recordings. Using the estimate of the dendritic receptive field size obtained from the morphological reconstruction, we plotted the ERFs along with the dendritic receptive fields for 21 cells (Fig 8C). Two cells where morphological reconstruction was possible were omitted due to uncertainty of the location of the cell relative to the array. The dendritic fields were estimated by a circle with a diameter equal to the major axis from the elliptical fit. The electrode colours represent the amplitude of ${\vec{w}}^{+}$ and the stars represent the approximate location of the optic disc. One cell (Fig 8C21) was only affected by cathodic-first stimulation and hence ${\vec{w}}^{-}$ is shown for this cell. This cell was affected by both anodic- and cathodic-first stimulation in its long-latency responses.

**Fig 9:**
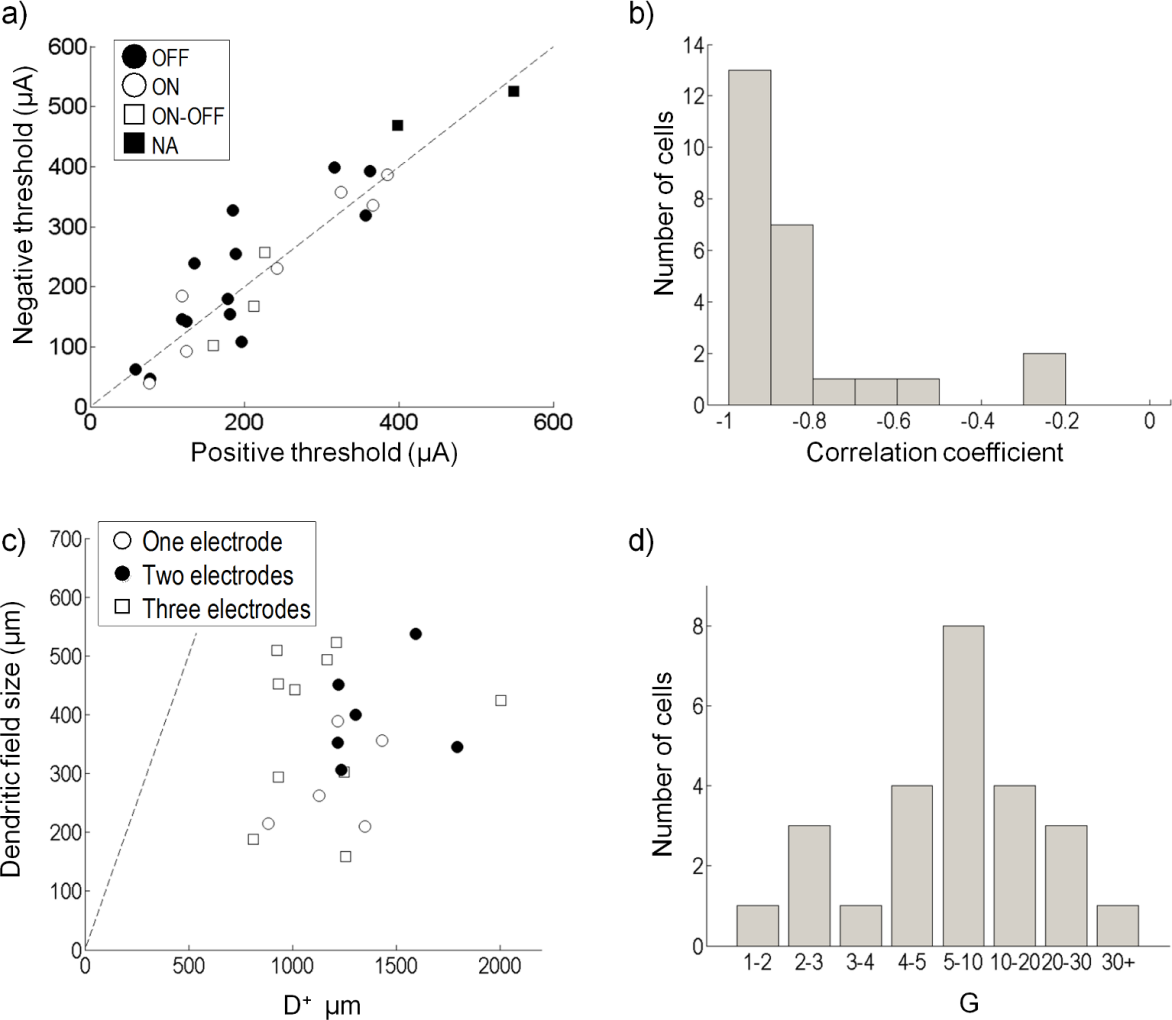
Population data. (a) Threshold recovered from the nonlinear function for positive and negative regions for all cells; no significant differences were found between cell types (p > 0.3). (b) The correlation coefficients of the positive and negative electrical receptive fields (${\vec{w}}^{+}$ and ${\vec{w}}^{-}$). (c) Radius of electrical influence for 21 cells. Shown is the approximate range of electrical influence for anodic-first stimulation (*D*^+^) and the dendritic field size for each cell. Cells were only significantly influenced by one (open circle), two (closed circle) or three (square) electrodes in the ERF. (d) Histogram showing the value of *G* (Eq (10)) for all cells. 20 of the 25 cells recovered a ${\vec{v}}_{1}$ that was 4 or more times larger than ${\vec{v}}_{2}$.

Data summarizing the model fit for the population of 25 cells is shown in Fig 9. The model nonlinear fit recovers an estimate of a cell’s thresholds (*c*_+_ and *c*_−_ in Eqs (6) and (7)). Most cells had similar threshold values for both their net anodic-first and net cathodic-first regions (see Table 1, Fig 9A), and no significant differences were found between or among ON, OFF or ON-OFF cell types (t-test, p > 0.3). The correlation coefficient of the ERFs ${,\vec{w}}^{+}$ and ${\vec{w}}^{-}$, for the majority of cells was close to -1 indicating that the cell was influenced by same electrodes for both net anodic-first and net cathodic-first stimulation (Fig 9B). Two OFF cells had a correlation coefficient greater than -0.4 suggesting that the cell was differentially influenced by the electrodes depending on whether the stimulus was anodic-first or cathodic-first. The size of the positive ERF (*D*^+^) was estimated for 23 cells and compared to the dendritic field size (Fig 9C). Cells were significantly influenced by only one (open circle), two (closed circle) or three (square) electrodes. The mean size of the ERFs produced by both ${\vec{w}}^{+}$ and ${\vec{w}}^{-}$ was approximately 1.2 mm, and no significant difference was found between the size produced by ${\vec{w}}^{+}$ and ${\vec{w}}^{-}$ (t-test, p>0.4).

The statistical hypothesis test showed that the spike-eliciting information was mostly contained in the first PCA component (${\vec{v}}_{1}$). For 17 cells, some information was also contained in up to two additional components; however this information was relatively small, as the effect of omitting these higher components made little difference to the predicted result. We used a ratio *G* measuring how much of the variance in the response is accounted for by the first principal component (${\vec{v}}_{1}$) compared to the next most significant component (${\vec{v}}_{2}$). *G* was generally much greater than one, signifying that for most cells a large proportion of the spike-triggered variance is contained in the first principal component. A histogram of *G* for all cells is given in Fig 9D, which shows that 18 of the 20 cells had a *G* value >4. Cells with a single dominant principal component have spatial interactions between electrodes that are linear to a good approximation.

### Model validation

**Fig 10:**
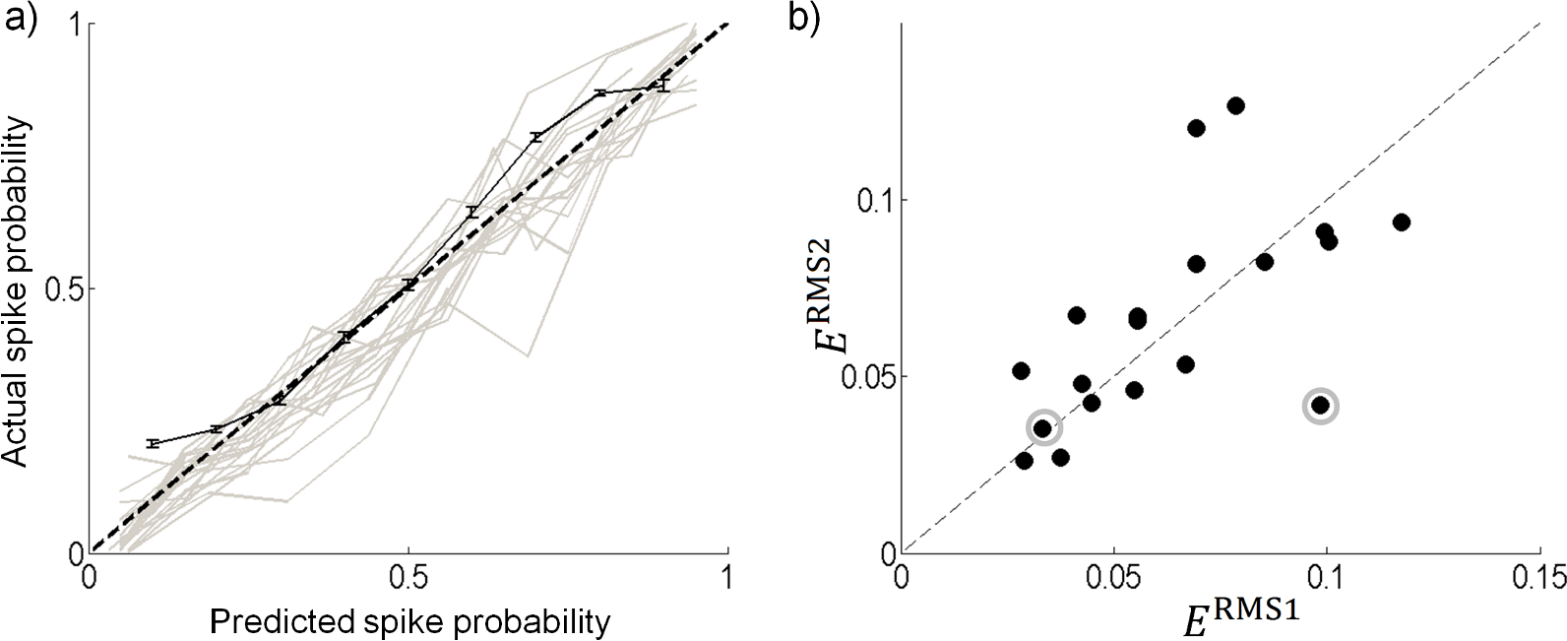
Model validation. (a) The predicted probability compared to the actual probability from the validation data for all cells (gray). For clarity this data is shown without error bars. The validation of the sample cell from Fig 7 with standard error bars (black solid line) is also shown. The root mean square error for this cell was 0.056. (b) The 2-dimensional model error (*E*^*RMS*2^) is compared to the 1-dimensional model error (*E*^*RMS*1^) for all cells where the hypothesis test recovered 2 or more significant components.

80% of the data was used to recover the model parameters and the remaining data was used to validate the model prediction. Fig 10A compares the validation spike probabilities and the model predicted probabilities for all cells (grey curves). For clarity, we show the curves without error bars. Also shown is the model prediction for one cell with standard error bars (black solid line). The model accurately predicted the responses of the RGCs to electrical stimulation. Small deviations in the prediction could likely be explained by modeling errors due to omission of some significant components. Using a different set of data to validate the model gave slightly different deviations, and slightly different estimates of the error. However, in all cases the model still accurately predicted the responses. The average *E*^RMS^ for all cells was 6.3% error, with a maximum error of 11.7% (see Table 1).

There were 17 cells that recovered two or more components that fell outside the 95% confidence interval in the statistical hypothesis test. To test for improvements in the prediction by including higher components, we fit a two-dimensional surface to the probability data (e.g. Fig 7A). The same validation data used from the one-dimensional model was used to compute the error from a two-dimensional model. Note that for some cells the training data was under-sampled in regions that were sampled with the validation data. These points were omitted when calculating the model error. The model error for the two-dimensional model (*E*^RMS2^) was compared to the error from the one-dimensional model (*E*^RMS1^). For most cells, little improvement was found in model error, and a few cells resulted in a higher error (Fig 10B). A slightly higher error for a few cells is likely due to over fitting to increased noise, which occurs due to undersampling of the two-dimensional surface fit. For the two cells that had a low value of *G*, the model error was reduced by approximately half (9.8% to 4.2%) for one cell and changed very little for the other (3.3% to 3.5%) (See the two circled points in Fig 10B).

### Long latency responses

**Fig 11:**
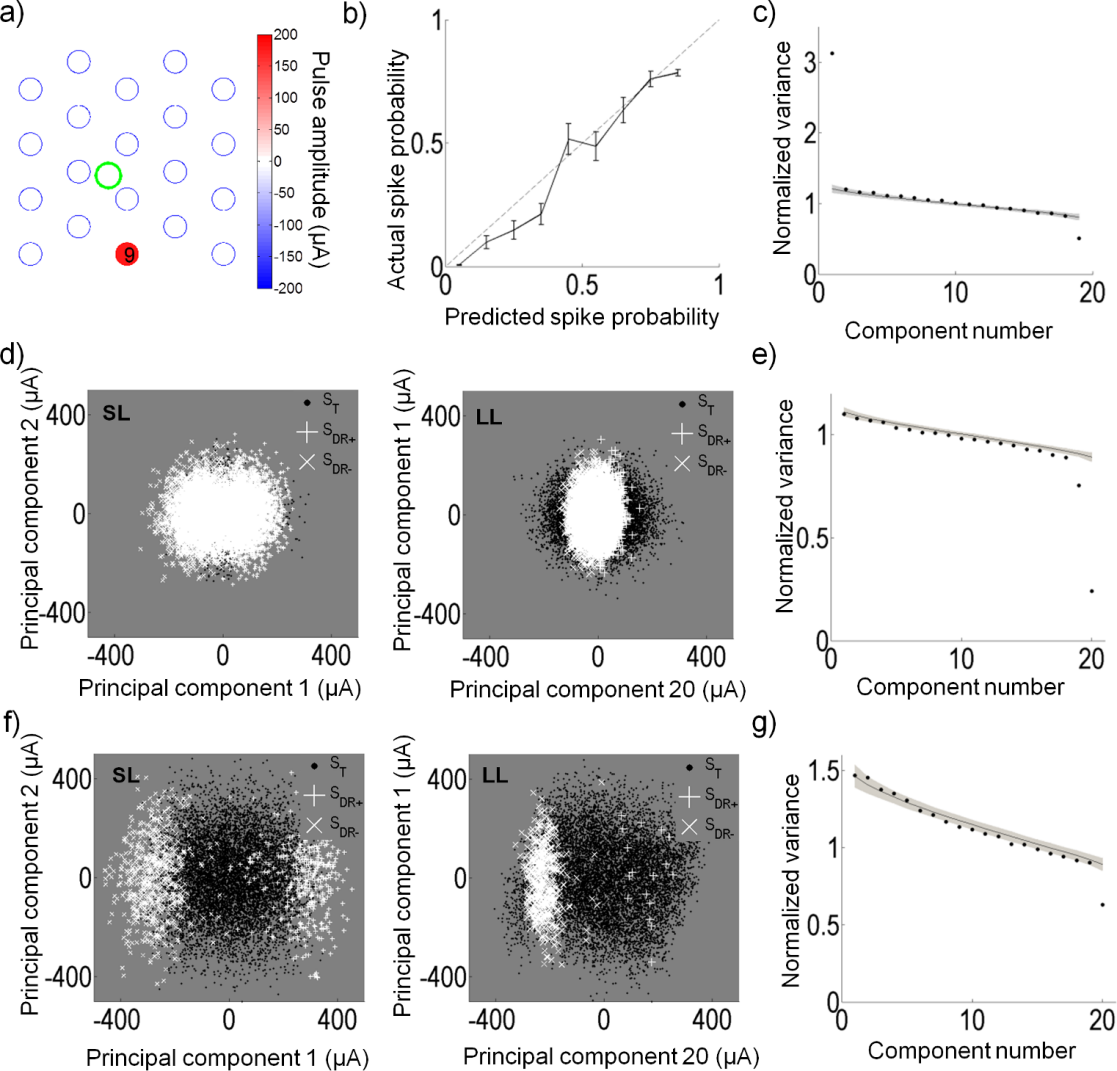
Model applied to long-latency responses. (a) The positive electrical receptive field for long-latency responses of the same cell as in Fig 7. This cell is largely influenced by electrode 9, which in this preparation was below the optic disc. (b) The predicted response vs. the actual response probability for the cell in (a). The root mean square error between prediction and actual response probability was 0.07. (c) The eigenvalues of the long-latency spike-triggered stimuli with one large excitatory and suppressive component evident. (d) An example of a cell with a suppressive response for long-latency (LL) responses. This cell fired with fewer spikes when the stimulus was stronger along the first or second principal component. Also shown is the short-latency (SL) response for comparison. (e) The corresponding eigenvalues for the long-latency responses in (d). (f) An example of a cell with a preferred stimulus polarity for long-latency responses. This cell responded with a greater number of spikes when the stimulus was cathodic-first, and very few spikes when the stimulus was anodic-first. Also shown is the short-latency response for comparison. (g) The corresponding eigenvalues for the long-latency responses in (f).

We examined if the model could also be applied to the long-latency responses to predict responses that were most likely of presynaptic network activity. Understanding the secondary effects of electrical stimulation is important in a clinical setting to understand differences between the perceived and expected responses. Responses originating from presynaptic origin can have excitatory or suppressive effects on postsynaptic RGCs. Fig 11A illustrates the positive ERF (${\vec{w}}^{+}$) for the sample cell (same cell from Fig 7) that had an excitatory long-latency response. The negative ERF was almost the same in magnitude and location as the positive ERF and hence is not shown. In this preparation, the optic disc was placed above electrode 9. It is this electrode that most strongly influences the long-latency response in this neuron. The accuracy of the model was assessed in the same way as the model error for the short-latency responses. For this cell, the model predicted the long-latency response accurately (error approximately 7%) (Fig 11B). It is evident from the corresponding eigenvalues (Fig 11C) that there is an excitatory and suppressive component that affects the long-latency responses. Excitatory or suppressive effects applied through the retinal network can be investigated by analysis of long-latency responses. Fig 11D illustrates an example of a cell that was very responsive in its short-latency responses; however, it became suppressed by high amplitude stimulation in its long-latency responses. The corresponding eigenvalues are shown in Fig 11E. Although our results on short-latency responses showed that RGCs were largely indifferent to the pulse polarity, analysis on the long-latency responses could produce responses that favored a particular pulse polarity. Fig 11F shows an example of a cell that was sensitive to both polarities of short-latency stimulation but its long-latency response resulted largely from cathodic-first stimulation. The corresponding eigenvalues are shown in Fig 11G.

### Application to efficient stimulation

**Fig 12:**
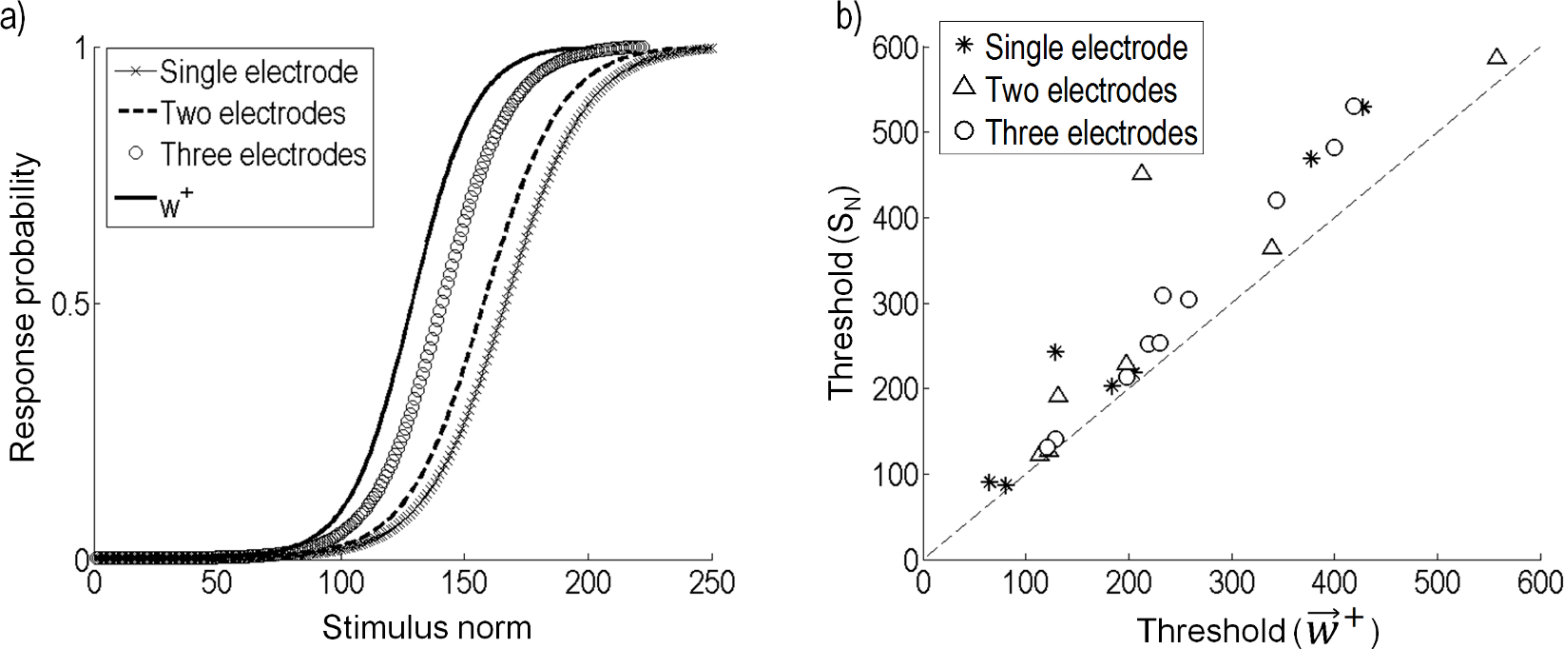
Comparison of stimulation along ${\vec{w}}^{+}$ to a multi-electrode, constant amplitude strategy. (a) The response probability when stimulating along ${\vec{w}}^{+}$ is compared to a naive strategy that uses only one electrode, or equal amplitude currents on two or three electrodes. (b) The naive strategy (S_N_) is compared to stimulating along ${\vec{w}}^{+}$. Only the S_N_ resulting in the lowest thresholds are shown; single electrode (star), two electrodes (triangle) or three electrodes (circle).

When little is known about the neural system, a naive stimulation strategy might be to activate multiple nearby electrodes such that the amplitude of stimulation across the electrodes is equal. However, the ERF recovered from the model gives an insight into the stimulus that improves the efficacy of a response in the neuron. To compare a naive stimulation strategy to stimulation using currents on electrodes that are proportional to ${\vec{w}}^{+}$, we used the recovered model to compare the response probabilities for both strategies. Fig 12A compares stimulation on one, two, or three of the electrodes closest to the sample cell, to stimulation with currents proportional to ${\vec{w}}^{+}$. To make an unbiased comparison, comparisons were made while keeping the total power fixed. Since all of the electrodes were of the same geometry, this was equivalent to keeping a constant norm on the stimulation vector $\vec{S_{t}}$. For this example, a stimulus on three of the closest electrodes, where the current amplitudes on each electrode were equal, resulted in better efficacy than stimulating on only one or two electrodes. However, the efficacy was further improved when stimulating proportionally to ${\vec{w}}^{+}$. Fig 12B compares the threshold from a naive strategy (S_N_), to the threshold of a stimulus proportional to ${\vec{w}}^{+}$ for all cells. Here, we compare only S_N_ resulting in the lowest threshold, single electrode (star), two electrodes (triangle), or three electrodes (circle). In all cases, stimulation proportional to ${\vec{w}}^{+}$ results in a higher efficacy for a given power. On average, stimulation proportional to ${\vec{w}}^{+}$ resulted in a threshold 0.8 (SD 0.2) times the threshold of the S_N_ resulting in the lowest threshold.

## Discussion

The research presented here is motivated by the goal to improve the fidelity of neural prostheses by improving stimulation strategies through the use of predictive models of neural response to electrical stimulation. The model we present describes a method to predict the response of neurons to patterns of concurrent electrical stimulation. It is simple to construct and evaluate, and could be applied clinically and throughout the nervous system.

### Mathematical model

The model we present here was adapted from well established Gaussian white noise models developed to describe light responses in the retina [16,18,20,22]. With minor modifications, we have shown the application of this type of model to describe responses to electrical stimulation. The model is scalable and can be used to also describe retinal responses to electrical stimulation using small, high density electrodes, or to describe long-latency responses.

RGC responses to concurrent electrical stimulation across multiple electrodes could be accurately modeled by a nonlinear transformation of a linear spatially filtered stimulus. Simultaneous biphasic pulses were applied to all electrodes, with the stimulation amplitude on each electrode randomly sampled from a Gaussian distribution. The model's linear filter characterizes the neuron's electrical receptive field. The nonlinear function characterizes the neuron's intrinsic nonlinear firing properties. Stimulus-evoked spikes in the recorded neurons were analyzed using principal component analysis to determine the linear filter and reduce the dimensionality of the spike-triggered stimuli.

For most cells a single linear filter was sufficient to predict the neural response to a good approximation, indicating that interactions between concurrently stimulated electrodes are predominantly linear. High coefficients of determination for the nonlinear function fits were obtained (lowest r^2^ = 0.83), demonstrating that the double sigmoid function is an accurate description of the nonlinearity. The model was trained with 80% of the data, with the remainder used for validation. The spike probability from the validation data was compared to the predicted probability, which resulted in an average error of 6.4% across the population (maximum error 11.7%).

For many cells, short-latency responses were within 2–3 ms from the stimulation offset. While the origin of the spikes were not investigated, the latencies are consistent with latencies attributed to direct activation of RGCs in response to 1 ms pulses [38]. Four cells showed overlapping short- and long-latency clusters with spike latencies of up to 6 ms (Fig 6D). It is possible that some of the spikes in the four cells with overlapping clusters might have had a mixture of direct and indirect (network mediated) activity. Despite this, the model was able to accurately predict the response. The technique could also be applied to investigate the long-latency responses driven by synaptic activity. The effects seen in the long-latency responses can be excitatory, suppressive (also observed in [38]), or polarity-selective. Importantly, a separate analysis of long-latency responses produced distinct electrical receptive fields compared to the short-latency responses. Long-latency responses in the retina are mediated via the activation of retinal interneurons and might result in high acuity vision [39]. The techniques described can be used to gain a deeper understanding of the retinal network and the effects of electrical stimulation at distant sites. Investigation into the long-latency responses can give insight into the secondary effects of stimulation and how these might influence perception.

### Comparison to previous studies

Models recovered from white noise stimuli have been used to characterize light responses in the retina [16,18,20] and cortex [40], electrical responses in the retina [26,41], subthreshold responses in squid axons [42], and to characterize information transfer from the sensory periphery [43]. The advantage of estimating models with Gaussian white noise is that the neurons can be presented with a wide range of possible inputs and adaptation is reduced compared to more regularly structured stimuli. These properties make these models more suitable for characterizing neural responses to spatiotemporal patterns of electrical stimulation. Additionally, analysis techniques for white noise stimulation have been extensively explored in the retina to describe light responses [16,18,20,21]. We have used a spike-triggered covariance model and demonstrated that it can be accurately applied to describe electrical responses.

Jepson et al. [44] demonstrated the versatility of a piecewise linear model in capturing neural response probabilities to electrical stimulation. Their study used a high-density array with small electrode diameters and combined stimulation across two or three electrodes to achieve spatial selectivity. Only fixed ratios of stimulus amplitudes were explored. In contrast, our stimuli consisted of Gaussian white noise that allowed the exploration of a vast range of stimulus inputs across all available electrodes, to find an estimate of the cell’s ERF. This has the potential to be more efficient when simultaneously recording from multiple neurons, as the same stimuli can be used to generate the model parameters for all recorded neurons. Our study also used large diameter electrodes due to their relevance to clinical visual prosthesis stimulation arrays [45].

No strong correlation between the area over which the cell was affected (*D*^+^ or *D*^−^), and the size of the dendritic field was found. It is possible that much of the relationship is lost when using large diameter electrodes, or that the stimulation was largely axonal. The spatial ERFs were generally as might be expected: i.e. the electrodes closest to the cell significantly affected the response. However, some unexpected results were apparent. For example, cells in Fig 8C17 and 8C18 both had a small dendritic field and they were located in a similar location in relation to the stimulating array. However, cell 17 only had two significant electrodes, whereas cell 18 had three. While the origin of these differences are unknown, complexities in ERF shapes have been previously observed [39,46]. As suggested by Sim et al. [46], the complex shapes could be due to axonal stimulation. Our results demonstrate the utility of our technique in identifying even complex ERFs.

Freeman et al. [26] explored single electrode stimulation of the retina using binary white noise to recover a temporal spike-triggered average stimulus. This study demonstrated that electrical white noise models can be used to estimate the temporal relationship between the stimulus and response, while our model describes the spatial relationship across electrodes. We assume that the effect of temporal interactions at 10 Hz is small, an assumption that might account for some of the error in prediction. A model that also incorporates temporal effects is desirable, especially when considering higher stimulation frequencies, but technical challenges remain. A similar model to Chichilnisky [16] could be modified for electrical stimulation and incorporate spatiotemporal ERFs, but the accuracy of the model would decrease as the number of frames increased. The stimulus artefact could also be a problem when stimulating at high frequencies. To obtain longer recordings, one solution is to record extracellular potentials, which is also the only practical solution for patient testing. In this case, the stimulus artefact would be larger than the spike signal, making spike identification more challenging. At low stimulus frequencies, the stimulus artefact could be removed by artefact removal techniques [39,47,48], allowing detection of short-latency spikes.

### Relevance for neural prostheses and clinical applications

A major goal of our work is to develop models that can be applied in real-time, closed-loop applications. The applications of closed-loop systems to modern technology are vast. There are several potential advantages to the development of neuroprostheses that make use of neural feedback. An obvious advantage of neural feedback is much tighter control of evoked neural activity, when that activity can be measured and the stimulus adjusted to match a desired outcome. A second advantage is automation of patient fitting procedures, minimizing the need for time-consuming psychophysics. Furthermore, many stimulation algorithms are limited to stimulation with one electrode at a time, in part because the time required to test myriad possible combinations of simultaneous electrode stimulation is prohibitive using a psychophysical approach. Closed-loop neural stimulation models can also take advantage of control theory and can be designed to adapt to changes in the system being controlled. Open-loop strategies cannot adapt to changes such as changes at the electrode-tissue interface.

The model we present is simple and appropriate for real-time computation; however, technical challenges remain. A requirement of *in vivo* or patient tests of closed-loop control is to obtain high density extracellular neural recordings. Devices that can combine stimulation and recording on the same electrode need to balance high surface area and charge capacity for stimulation, with electrodes of low geometric surface area for single-unit recordings [45]. Recent new materials have led to the development of flexible electrode wires capable of stimulation and recording [49]. However, a high density array capable of stable recordings and stimulation remains to be developed.

Our model can be extended by increasing the number of recording electrodes, to describe the response of multiple neurons across the array, thus making it a multi-input multi-output system. Multi-input multi-output control is a widely researched area of control and could be applied to achieve patterns of activation across the array. In a visual prosthesis, a desired pattern of activation could be obtained from RGC activation models in response to patterned light [16,50]. Our model can be fit to individual patients based on recorded responses and used to develop control strategies that are patient specific. Devices that can record and stimulate can then be used to try and address some of the more complex problems in the field, namely that of relating stimulation to visual percept [51].
